# Electrochemical sensor for rapid determination of fibroblast growth factor receptor 4 in raw cancer cell lysates

**DOI:** 10.1371/journal.pone.0175056

**Published:** 2017-04-04

**Authors:** Rebeca M. Torrente-Rodríguez, Víctor Ruiz-Valdepeñas Montiel, Susana Campuzano, María Pedrero, Meryem Farchado, Eva Vargas, F. Javier Manuel de Villena, María Garranzo-Asensio, Rodrigo Barderas, José M. Pingarrón

**Affiliations:** 1 Departamento de Química Analítica, Facultad de CC. Químicas, Universidad Complutense de Madrid, Madrid, Spain; 2 Departamento de Bioquímica y Biología Molecular, Facultad de CC. Químicas, Universidad Complutense de Madrid, Madrid, Spain; Kermanshah University of Medical Sciences, ISLAMIC REPUBLIC OF IRAN

## Abstract

The first electrochemical immunosensor for the determination of fibroblast growth factor receptor 4 (FGFR4) biomarker is reported in this work. The biosensor involves a sandwich configuration with covalent immobilization of a specific capture antibody onto activated carboxylic-modified magnetic microcarriers (HOOC-MBs) and amperometric detection at disposable carbon screen-printed electrodes (SPCEs). The biosensor exhibits a great analytical performance regarding selectivity for the target protein and a low LOD of 48.2 pg mL^-1^. The electrochemical platform was successfully applied for the determination of FGFR4 in different cancer cell lysates without any apparent matrix effect after a simple sample dilution and using only 2.5 μg of the raw lysate. Comparison of the results with those provided by a commercial ELISA kit shows competitive advantages by using the developed immunosensor in terms of simplicity, analysis time, and portability and cost-affordability of the required instrumentation for the accurate determination of FGFR4 in cell lysates.

## Introduction

Nowadays cancer is one of the main causes of death worldwide [1]. In fact, cancer mortality is becoming close to that of heart attacks [2]. As a consequence, there is a high interest on the development of efficient strategies to diagnose the disease and fight against it. Early detection of neoplastic tumors is important due to the difficulty of combating cancer in advanced stages. Moreover, together with reliable cancer diagnosis, discovering new anticancer drugs and treatments is not less important.

Monitoring cancer biomarkers is at present among the most popular methods for cancer diagnosis. Cancer biomarkers include nucleic acids, secreted proteins, and small molecule metabolites present in blood and saliva. In particular, monitoring of cancer biomarker proteins, which have been identified for every major type of cancer, is considered promising for early detection and therapy and surgery monitoring [3].

In this context, fibroblast growth factors (FGFs) and their tyrosine kinase receptors (FGFRs) are relevant in various biological processes through regulation of pathways involved in mitogenesis, proliferation, differentiation, development, angiogenesis, tumorigenesis and survival [4, 5]. FGF receptors dysregulation occur through overexpression, gene amplification or mutation and play an important role in cancer development and progression [6, 7]. In particular, alterations in fibroblast growth factor receptor 4 (FGFR4) occur in breast, ovarian, prostate, colon, rhabdomyosarcoma, pancreatic, gastric, hepatocellular and pituitary adenocarcinomas [4–6, 8]. Moreover, FGFR4 overexpression is associated with metastasis, late stages and poor prognosis in colorectal, gastric, lung, breast adenocarcinoma, rhabdomyosarcoma and high-grade serous ovarian carcinoma [6, 8, 9] and probably contribute to the progression of cancer by regulating proliferation and anti-apoptosis. Up to date, no sensor, either optical or electrochemical, has been described for the determination of FGFR4, which is mainly detected by immunohistochemistry or Western Blot [4–6, 9, 10]. In general, the most popular methods for cancer diagnosis include biopsy analysis, tumor imaging, enzyme-linked immunosorbent assays (ELISA), and mass spectrometry-based proteomics [2]. Methodologies using biosensing strategies are currently being developed as promising alternatives to those laborious, expensive, and long-lasting procedures. In this context, the use of electrochemical immunosensors constitutes an interesting solution due to their inherent high sensitivity and selectivity, precision and accuracy, relatively low cost, minimum sample requirement, simplicity of operation and possible integration in compact analytical devices [11]. In particular, sandwich immunoassays involving the attachment of capture antibodies on magnetic microcarriers, which allow a highly efficient capture and separation of the biomarker target protein from the liquid samples and electrochemical detection at disposable electrodes [11–18] have attracted a wide interest in recent years. In this paper, the first electrochemical immunosensor, involving a rapid and simple methodology, for the determination of FGFR4 in cell lysates is described. The protein is selectively captured on micro-sized magnetic beads and further sandwiched with a secondary HRP-labelled antibody. The formed immunocomplexes were magnetically captured and amperometrically detected at screen-printed carbon electrodes (SPCEs) using the hydroquinone (HQ)/H_2_O_2_/horseradish peroxidase (HRP) system.

## Materials and methods

### Apparatus and electrodes

Amperometric measurements were performed with a CHI812B potentiostat (CH Instruments, Austin, TX, USA) controlled by software CHI812B. Screen-printed carbon electrodes (SPCEs) (DRP-110, DropSens, Llanera (Asturias), Spain), consisting of a 4-mm diameter carbon working electrode, a carbon counter electrode and an Ag pseudo-reference electrode, were used as electrochemical transducers together with a specific cable connector (DRP-CAC, DropSens, S.L.), which acted as interface between the SPCEs and the potentiostat.

A Bunsen AGT-9 Vortex (Velp Scientifica, Usmate, MB, Italy) was employed for homogenization of the solutions. A Thermomixer MT100 constant temperature incubator shaker (Universal Labortechnik, Leipzig, Germany), a magnetic separator DynaMag-2 Magnet (ThermoFisher Scientific,Waltham, MA, USA)) and a Magellan V 7.1 (TECAN, Männedorf, Switzerland) ELISA plate reader were also employed. Once MBs were conveniently modified, they were captured onto the surface of the SPCEs and controlled by a neodymium magnet (AIMAN GZ) embedded in a homemade Teflon casing. All measurements were performed at room temperature.

### Reagents and solutions

All reagents used were of the highest available analytical grade. Carboxylic acid-modified magnetic beads (HOOC-MBs, 2.7 μm Ø, 10 mg mL^-1^, Dynabeads^®^ M-270 carboxylic acid, Cat. No: 14305D) were purchased from Invitrogen-Thermo Fisher (Waltham, MA, USA). 2-(N-morpholino)ethanesulfonic acid (MES), sodium chloride, potassium chloride, sodium di-hydrogen phosphate, di-sodium hydrogen phosphate and Tris-hydroxymethyl aminomethane-HCl (Tris-HCl) were purchased from Scharlab (Barcelona, Spain). *N*-(3-dimethylaminopropyl)-*N’*-ethylcarbodiimide (EDC), *N*-hydroxysulfosuccinimide (Sulfo-NHS), ethanolamine, hydroquinone (HQ), Tween^®^ 20 and hydrogen peroxide (H_2_O_2_) (30%, w/v) were purchased from Sigma-Aldrich (St. Louis, MO, USA). Commercial blocker casein solution (a ready-to-use, PBS solution of 1% w/v purified casein) was purchased from Thermo Scientific (Thermo Fisher, Waltham, MA, USA). Mouse anti-human FGFR4 antibody (CAb), biotinylated rat anti-human FGFR4 antibody (BDAb), and recombinant human FGFR4, as components of the Human Total FGFR4 DuoSet^®^ IC ELISA kit from (from R and D Systems, Inc., Minnneapolis, MN, USA, Catalog Number DYC685-2) were used. A high sensitive streptavidin-peroxidase (Strep-HRP) conjugate from Roche (Ref 000000011089153001 from Sigma Aldrich, St. Louis, MO, USA) was employed as enzymatic label. Bovine serum albumin (BSA Type VH) from Gerbu Biotechnik (Heidelberg, Germany, GmbH), human hemoglobin (Sigma-Aldrich, St. Louis, MO, USA, H7379) and IgG from human serum (Sigma-Aldrich, St. Louis, MO, USA, I2511), recombinant human TNFα protein (in TNF-alpha EIA Kit, ELISA, RUO, 12 × 8 wells, Beckman Coulter, Alcobendas, Madrid, Spain, ref. IM1121) from Immunotech and N-terminal GST-tagged, recombinant full length human p53 protein (Catalog#14–865) from EMD Millipore Corporation (Merck Millipore, Barcelona, Spain), were tested as potential interfering compounds.

All the required buffer solutions were prepared with water from Millipore Milli-Q purification system (18.2 MΩ cm): 0.05 M phosphate buffer, pH 6.0; 0.1 M phosphate buffer, pH 8.0; phosphate-buffered saline (PBS) consisting of 0.01 M phosphate buffer solution containing 0.137 M NaCl and 0.0027 M KCl, pH 7.5; 0.025 M MES buffer, pH 5.0; 0.01 M sodium phosphate buffer consisting of PBS supplemented with 0.05% Tween ^®^ 20 (PBST, pH 7.5) and 0.1 M Tris-HCl buffer, pH 7.2.

An EDC/sulfo-NHS mixture solution (50 mg mL^-1^ each, prepared in MES buffer, pH 5.0) and a 1 M ethanolamine solution (prepared in 0.1 M phosphate buffer solution, pH 8.0) were used for activation and blocking steps of the HOOC-MBs, respectively.

### Preparation of the MBs-based electrochemical immunosensor

Before removing the supernatant and after all incubation/washing steps (performed at 25°C), eppendorfs containing MBs were placed in the magnetic separator and concentrated for 4 min.

The optimized procedure for the biofunctionalization of the magnetic micro-carriers was carried as follows: a 3 μL-aliquot of the HOOC-MBs commercial suspension was deposited into a 1.5 mL Eppendorf^®^ tube and washed twice with 50 μL of MES buffer for 10 min at 25°C and under continuous stirring (950 rpm). Thereafter, carboxyl groups of the magnetic micro-carriers were activated by incubation in 25 μL of a freshly EDC/sulfo-NHS solution (50 mg mL^-1^ each, in MES buffer, pH 5.0) for 35 min (25°C, 950 rpm) and, after two washing steps with MES buffer, the microbeads were incubated for 15 min (25°C, 950 rpm) in 25 μL of a 5.0 μg mL^-1^ CAb solution (prepared in MES buffer). Next, CAb-MBs were washed two times with MES buffer, pH 5.0, and incubated for 1 h (25°C, 950 rpm) in a 1 M ethanolamine solution (prepared in 0.1 M phosphate buffer, pH 8.0) for blocking the remaining unreacted and previously activated carboxyl groups of the micro-carriers. After the blocking step, the CAb-MBs were washed once with 0.1 M Tris-HCl buffer, pH 7.2 and twice with the commercial blocker casein solution, and subsequently incubated for 15 min (25°C, 950 rpm) in a 25 μL aliquot of a mixture solution containing a variable concentration of the FGFR4 standard (or the sample to be analyzed) supplemented with 1.0 μg mL^-1^ of BDAb and 1/1,000 Strep-HRP enzymatic conjugate (prepared in commercial blocker casein solution). In this way, sandwich immunocomplexes of HRP-Strep-BDAb-FGFR4-CAb were attached to the surface of the micro-carriers.

Finally, the as-modified magnetic microcarriers were washed twice with PBST, pH 7.5, and re-suspended in 50 μL of 0.05 M phosphate buffer, pH 6.0, to perform the amperometric measurements.

For the preparation of non-CAb-modified MBs similar activation and blocking protocols used for the preparation of Cab-modified MBs were employed but omitting the addition of the CAb in the MES buffer.

### Amperometric measurements

Amperometric measurements were performed as described in detail previously [13, 19]. Briefly, 50 μL of modified MBs suspension were pipetted onto the surface of the SPCE working electrode previously placed in a homemade Teflon casing with a neodymium magnet encapsulated. Then, the ensemble SPCE/magnet holding block was immersed into an electrochemical cell containing 10.0 mL of 0.05 M phosphate buffer, pH 6.0, and 1.0 mM HQ (prepared just before the electrochemical measurement). Amperometric readouts were recorded by applying a detection potential of −0.20 V (*vs*. the Ag pseudo-reference electrode) in stirred solutions upon addition of 50 μL of a 0.1 M H_2_O_2_ solution until reaching the steady-state (approx. 100 s). The amperometric signals given through the manuscript corresponded to the difference between the steady-state and the background currents. Unless otherwise stated, the presented data corresponded to the average of at least three replicates (confidence intervals calculated for α = 0.05).

### Cell culture and lysate production

SW480, SW620, MCF-7, MDA-MB-436 and BxPC3 cell lines, from the American Type Culture Collection (ATCC) cell repository, and KM12C and KM12SM cell lines (from I. Fidler's laboratory (MD Anderson Cancer Center, Houston, TX), were grown according to established protocols in Dulbecco’s modified Eagle’s medium (DMEM), supplemented with 10% fetal bovine serum, penicillin and streptomycin, and 2.5 mM L-glutamine (GIBCO-Invitrogen, Carlsbad, CA, USA) supplemented with recommended nutrients.

Cell lysis procedure was performed as follows: cells were washed with cold PBS, incubated for 5 min with PBS 4 mM EDTA to detach them from the plates, and centrifuged at 1,200 rpm to remove PBS 4 mM EDTA. Then, cells were re-suspended in 1 mL cold lysis buffer (25 mM Tris-HCl, pH 7.6, 150 mM NaCl, 1% NP-40, 1% sodium deoxycholate, 0.1% SDS) supplemented with 1X protease inhibitor cocktail, 1mM phenylmethylsulfonyl fluoride, and 1 mM activated sodium orthovanadate. Thereafter, cells were incubated on ice for 10 min, passed through a 25 gauge needle attached on a 1 mL syringe for 10 times and transferred to a microcentrifuge tube and centrifuged at 13,200 rpm at 4°C for 15 min. Total protein concentration was estimated using a BCA protein assay kit (Pierce, Rockford, IL) and, additionally, qualitative analyzed by Western Blot. Resultant cell lysates were stored at −80°C.

### Sodium Dodecyl Sulfate-Polyacrylamide (SDS-PAGE) and immunodetection analysis

10 μg of each protein extract were run in 10% sodium dodecyl sulfate-polyacrylamide gel electrophoresis (SDS-PAGE) and transferred to nitrocellulose membranes (Hybond-C extra) using semi-dry transfer (Bio-Rad) [20, 21]. After blocking, membranes were incubated at optimized dilutions with alternatively BDAb or RhoGDI policlonal antibody (SCBT, Ref. sc-33201,0.2 mg/ml, 1:1000) as loading control and followed by incubation with Strep-HRP (Raybiotech, AA-HRP-C, Lot#C 09122815) or secondary goat anti-rabbit IgG HRP labeled (BioRad, Ref. 1706515) at 1/3000 dilution, respectively. Specific reactive proteins were visualized with SuperSignal West Pico Maximum Sensitivity Substrate (Pierce).

### Application to the analysis of cell lysates

The usefulness of the developed sensor was demonstrated by analyzing the endogenous content of FGFR4 in different cell lysates expressing variable levels of the target receptor.

After verifying the absence of matrix effect in diluted samples (up to a final amount of 2.5 μg of raw lysate), CAb-MBs, prepared as described in the *“Preparation of the MBs-based electrochemical immunosensor”* Section, were re-suspended in a 25 μL aliquot of the appropriately diluted sample supplemented with 1.0 μg mL^-1^ BDAb and 1/1,000 Strep-HRP enzymatic conjugate (prepared in commercial blocker casein solution) and incubated for 15 min (25°C, 950 rpm). Then, the endogenous concentration of FGFR4 was determined just by interpolation of the recorded amperometric signals into the calibration plot constructed using FGFR4 standards. The reliability of the results provided with the developed electrochemical sensor was evaluated by analyzing the same samples with an ELISA method using the same immunoreagents.

## Results and discussion

**Fig 1:**
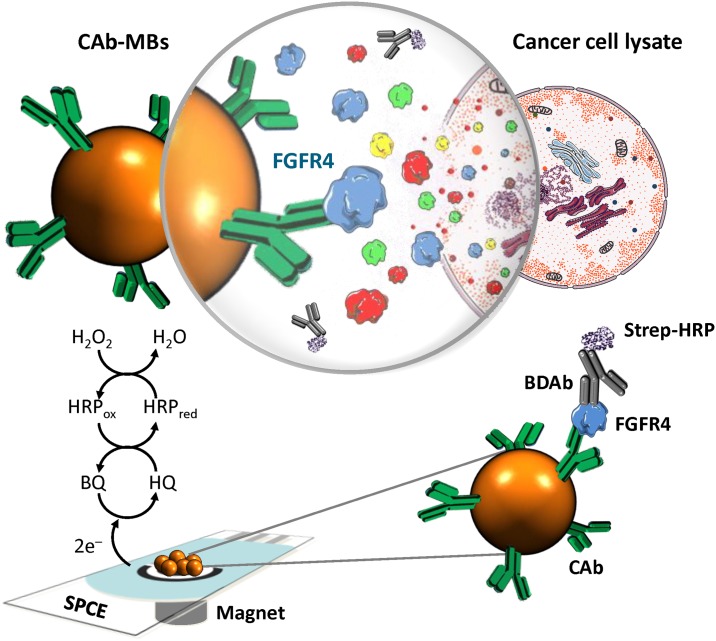
Schematic representation of the amperometric sensor for FGFR4 determination using magnetic immunocarriers and a sandwich format.

The fundamentals and the electrochemical immunosensor developed for the determination of FGFR4 are displayed in Fig 1. Briefly, magnetic immunocarriers were employed for the selective capture of the target protein and to implement a sandwich format using a biotinylated detector antibody coupled to an enzymatic polymer (Strep-HRP). The reactions involved in the amperometric quantification of the FGFR4 protein at SPCEs using the HQ/H_2_O_2_ redox system are also shown in Fig 1.

### Optimization of the experimental variables

The feasibility of the sandwich configuration and the efficiency of the blocker casein solution to minimize unspecific adsorptions on the MBs [22], were demonstrated by comparing the amperometric responses measured with immunosensors constructed with CAb-immobilized or non-CAb-modified MBs in the absence and in the presence of 10,000 pg mL^-1^ FGFR4. Results obtained demonstrated negligible bindings of FGFR4, BDAb and Strep-HRP in the absence of immobilized CAb on activated and blocked HOOC-MBs (data shown in S1 Fig), thus demonstrating the possibility to perform the FGFR4 determination using a sandwich format. In addition, all the experimental variables involved in the immunosensor preparation and functioning were optimized by measuring the amperometric responses obtained at −0.20 V (*vs*. the Ag pseudo-reference electrode) in the absence (B) and in the presence (S) of 5,000 pg mL^-1^ of FGFR4. The resulting (S/B) ratio was evaluated as the selection criterion for each experimental variable tested. The detection potential was previously optimized for the HRP/HQ/H_2_O_2_ system [23]. Table 1 summarizes the checked variables, the ranges into which they were measured and the selected values for each of them.

**Table 1:** Optimization of the experimental variables tested, according to the measured S/B ratio in the absence and in the presence of 5,000 pg mL^-1^ FGFR4 standard solutions, for the preparation of the amperometric immunosensor for FGFR4.

Variable	Checked range	Selected value
HOOC-MBs, μg	10–50	30
[CAb], μg mL^-1^	0–10	5.0
CAb_incubation time_, min	15–60	15
[BDAb], μg mL^-1^	0–2.5	1.0
Steps of the immunoassay	1–3	1
Incubation time_1 step_, min	15–60	15
Strep-HRP dilution factor	1/250–1/5,000	1/1,000

**Fig 2:**
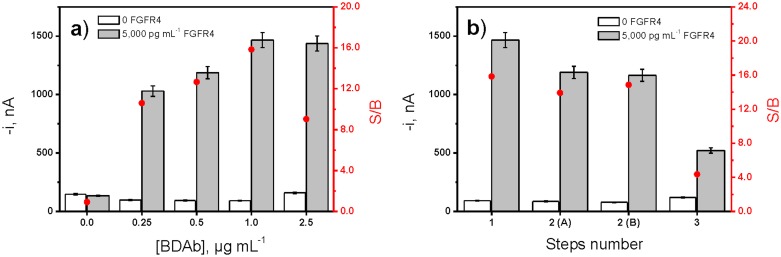
Dependence of the amperometric responses obtained with the developed immunosensor with the BDAb concentration (a), and the number of steps carried out to perform the immunoassay (b). Results are shown in the absence (white bars) or in the presence of 5,000 pg mL^-1^ FGFR4 (grey bars) together with the corresponding S/B ratio (•). 2(A), two sequential steps involving 30 min incubation of the CAb-MBs in a mixture solution containing FGFR4 and BDAb, and 30 min incubation in the Strep-HRP solution; 2(B) two steps involving 30 min incubation of the Cab-MBs with FGFR4 solution, followed by a 30 min incubation step in a mixture solution containing BDAb and Strep-HRP. Error bars estimated as triple of the standard deviation (n = 3).

As representative examples of all the optimized experimental variables, Fig 2 shows results obtained in the optimization of the BDAb concentration and the steps number to perform the immunoassay.

Fig 2a shows as the S/B ratio increased with the concentration of the BDAb up to 1.0 μg mL^-1^ then decreasing for larger concentration mainly due to an increase in the background current measured in the absence of the target protein a slight diminution of the signal current (in the presence of FGFR4) for higher CAb loadings due to the sterically hindered binding of the antigen when high amounts of capture antibody are immobilized [18]. Regarding the optimization of the steps involved in the immunoassay, different working protocols were assayed: 1) a single step involving both the capture of the target antigen and its labeling by means of 30 min incubation of the CAb-MBs in a mixture solution containing FGFR4, BDAb and Strep-HRP; 2A) two sequential steps involving 30 min incubation of the CAb-MBs in a mixture solution containing FGFR4 and BDAb followed by 30 min incubation in the Strep-HRP solution; 2B) two steps involving 30 min incubation of the CAb-MBs in the FGFR4 solution, followed by a 30 min incubation step in a mixture solution containing BDAb and Strep-HRP; and 3) three sequential steps involving 30 min incubation steps in FGFR4, BDAb and Strep-HRP solutions, respectively. Fig 2b shows as better S/B ratios were obtained by applying a single incubation step probably due to a better efficiency of the immuno- and labeling reactions when all the reagents are free in homogeneous solution. It is important to mention that, apart from a better performance, the use of a 1-step immunoassay simplified considerably the analysis protocol also implying a significant reduction in the assay time. As it is shown in Table 1, the incubation time for this single step was also optimized and 15 min was selected for further studies. Results achieved in the optimization of other experimental variables, summarized also in Table 1, are displayed in S2 Fig.

### Analytical characteristics

The amperometric measurements provided by 6 different immunosensors prepared in the same manner the same day for 2,500 pg mL^-1^ FGFR4 yielded a relative standard deviation (RSD) value of 4.4%, indicating the good reproducibility of the whole procedure including both the sensor fabrication (immune and labeling reactions onto magnetic microcarriers and their magnetic capture on the SPCE surface) and the amperometric transduction.

**Fig 3:**
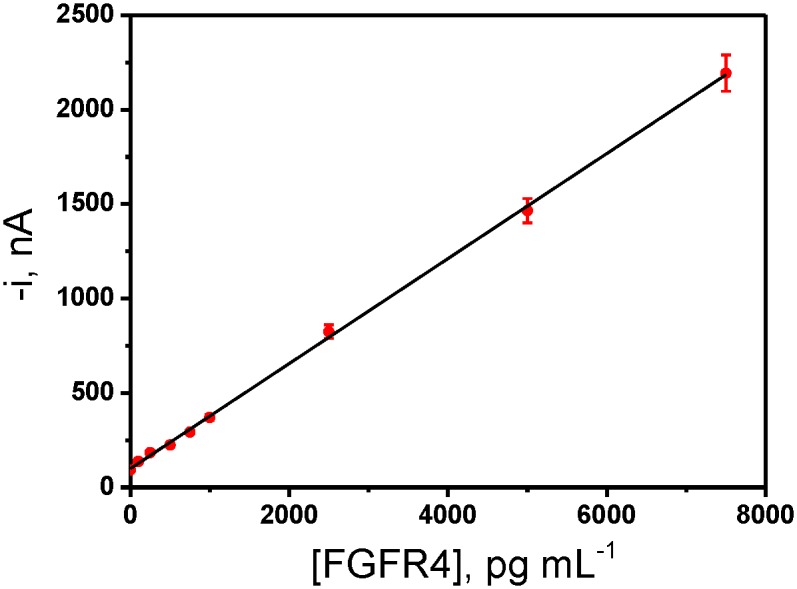
Dependence of the amperometric response measured with the developed magnetic immunocarriers-based sensor with the concentration of FGFR4 standards. Error bars estimated as triple of the standard deviation (n = 3).

Under the optimized working conditions, the calibration plot shown in Fig 3 was constructed with FGFR4 standards. A linear relationship (r = 0.999) was obtained over the 160.6–7,500 pg mL^-1^ FGFR4 concentration range with slope and intercept values of (0.278 ± 0.002) nA mL pg^-1^ and (99 ± 8) nA, respectively. The limits of detection (LOD) and quantification (LQ) achieved with the developed methodology were 48.2 and 160.6 pg mL^-1^, respectively. These values were estimated according to the 3×s_b_ /m criterion, where s_b_ was estimated as the standard deviation of 10 amperometric signals measured in the absence of FGFR4 and m is the slope value of the calibration graph.

In order to evaluate the storage stability of the CAb-modified magnetic microcarriers, different batches of CAb-MBs were prepared and stored at 4°C in Eppendorf tubes containing 50 μL of filtered PBS (pH 7.5). Every testing day, the amperometric responses provided by two different batches of the stored conjugates after incubation in solutions containing 0.0 and 2,500 pg mL^-1^ FGFR4 were measured following the procedures described in sections 2.3 and 2.4. Control charts were constructed for S/B values taking the mean value of 10 measurements obtained the first day of this study (day of AbC-MBs preparation) as the central value and ±3×SD of this initial value as upper and lower control limits. Results obtained (S3 Fig), showed that the S/B values remained within the control limits for a period of 15 days, suggesting that AbC-MBs could be prepared and stored for 2-weeks until the determination was required.

The lack of sensors described so far for the determination of FGFR4 only allowed us to compare these analytical characteristics with those of commercial ELISA methodologies. At this point, it is important to remark that although the sensitivity of the developed sensor is similar to that claimed for the commercial ELISA spectrophotometric kit (5.0 vs 3.1 pmol) using the same immunoreagents, an important advantage of the electrochemical immunosensor is that the analysis can be made in a much shorter period of time (15 vs 280 min, once the AbC-MBs and AbC-plate were prepared and blocked, respectively). In addition, the assay protocol is remarkably simpler, requiring only one incubation step in the sample solution supplemented with BDAb and Strep-HRP, which together with the involvement of portable and cost-effective instrumentation makes this electrochemical assay more easily automated and miniaturized. These characteristics make the developed MBs-based immunosensor an attractive and user-friendly tool to perform decentralized analysis or to monitor FGFR4 levels routinely.

### Selectivity

The selectivity of the developed immunosensor towards the target receptor was evaluated by checking various non-target proteins (TNFα, p53, BSA, hemoglobin and human IgG) as negative controls. It is important to mention here that as described in the specifications of the commercial ELISA kit, the antibodies used in the immunosensor did not cross-react with the other three members of the FGFR family (FGFR1, FRGR2 and FGFR3), reason why their interference was not evaluated in this study.

**Fig 4:**
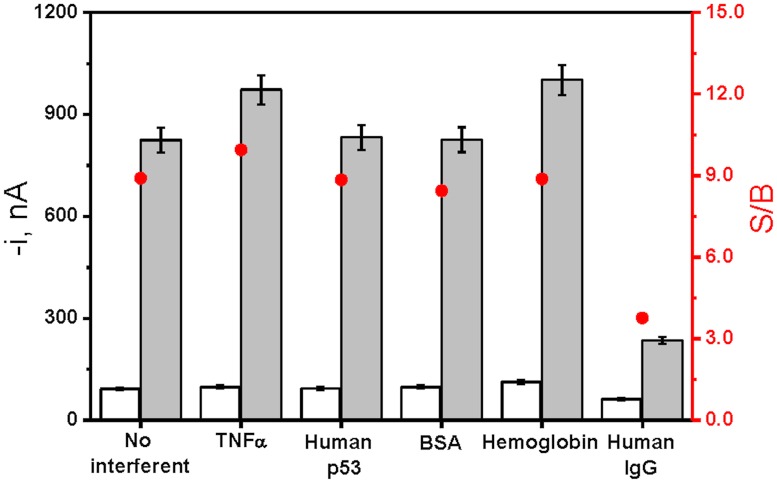
Evaluation of the selectivity of the developed immunosensor for FGFR4. Amperometric signals measured in the absence (white bars) and in the presence of 2,500 pg mL^-1^ FGFR4 (grey bars) and the corresponding S/B ratio (•) in the absence (1) and in the presence of 10 ng mL^-1^ TNFα (2), 200 ng mL^-1^ human p53 (3), 5 mg mL^-1^ BSA (4), 5 mg mL^-1^ hemoglobin (5) and 1 mg mL^-1^ human IgG (6). Error bars estimated as triple of the standard deviation (n = 3).

This selectivity study implied the comparison of the amperometric responses measured with the immunosensor for 0.0 and 2,500 pg mL^-1^ FGFR4 solutions prepared in the absence and in the presence of the potentially interfering compounds at their respective common concentrations found in comon biological samples like serum: 10 ng mL^-1^ TNFα; 200 ng mL^-1^ p53; 5 mg mL^-1^ BSA; 5 mg mL^-1^ hemoglobin and 1 mg mL^-1^ human IgG. Results displayed in Fig 4 show as similar S/B ratios were obtained in the absence and in the presence of TNFα, p53, BSA, and hemoglobin, thus indicating that no significant interference of these non-target proteins occurred in the FGFR4 determination. However, a dramatic reduction in the S/B ratio was observed in the presence of 1 mg mL^-1^ human IgG (bar 6 in Fig 4) which can be attributed to the presence of human anti-mouse antibodies (HAMAs) or to the three immunoglobulin-like domains of FGFR4 [24] which would lead to significant errors in sandwich immunoassays using mouse monoclonal antibodies, since they can cross-link the capture and labeled antibodies in the absence of the analyte [25]. However, it is important to mention that the quantification of the target receptor at a 2,500 pg mL^-1^ level was still feasible in the presence of such high concentration of human IgG. It is worth to mention at this point that, currently, there are some commercially available solutions that have demonstrated to be efficient in eliminating this human IgG widely reported interference in commercial diagnostic sandwich immunoassays based on the use of mouse monoclonal antibodies. Among them, we can point out the use of an IgG absorbent, which is a purified goat anti-human IgG Fc fragment (GAH IgG Fc) designed for the removal of human IgG from serum prior to testing, or a specific blocker called “TRU Block” which, after binding to the interfering antibodies, prevents further binding of heterophillic antibodies to other assay components through steric hindrance.

## Application to the determination of the endogenous FGFR4 in cell lysates

The proposed methodology was applied to the determination of FGFR4 in raw lysates from 8 different cancer cells expressing variable amounts of the target receptor. Cancer cells from breast (MCF7, MDA-MB-436 and SKBR3), colon (KM12C, KM12SM, SW480 and SW620) and pancreas (BxPC3) were selected for the study. The complexity and the small amount of target protein contained in these samples required a high sensitivity and selectivity in the employed methodology, features demonstrated by the developed sensor, to provide reliable determinations.

**Fig 5:**
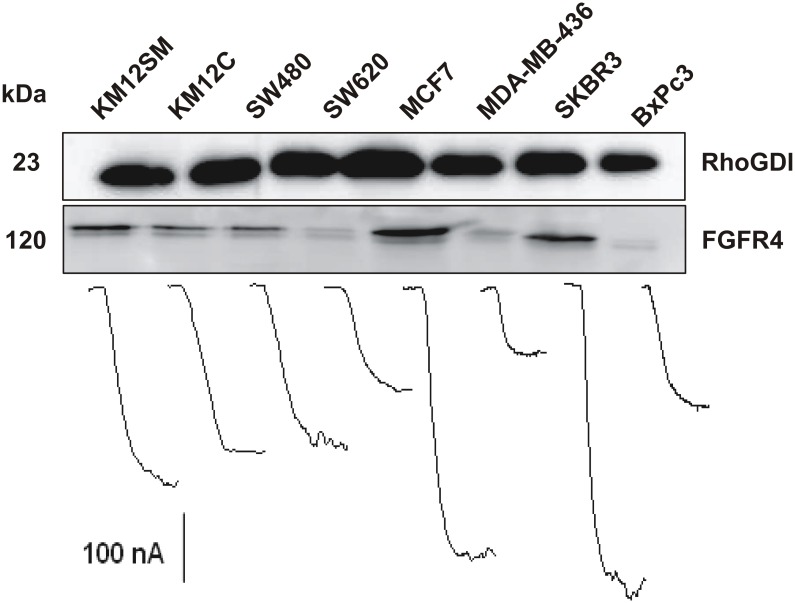
Analysis of FGFR4 in cell lysates by Western Blot (10 μg) and amperometric traces recorded with the developed immunosensor (2.5 μg).

No statistically significant differences were found between the slope value of the calibration plot constructed with FGFR4 standards (Fig 3, (0.278 ± 0.002) nA mL ng^−1^) and the similar slope values of the calibration graphs constructed for samples solutions containing 2.5 μg of raw lysates solution supplemented with increasing amounts of a standard FGFR4 solution up to 5,000 pg mL^-1^ ((0.27 ± 0.04) nA mL ng^−1^ as representative example using KM12SM lysates, S4 Fig). Therefore, the endogenous FGFR4 concentration in the lysates could be determined in a straightforward manner by interpolating the amperometric responses measured for the diluted samples (up to a final lysate amount of 2.5 μg) into the calibration plot prepared with FGFR4 standards. Fig 5 displays both the qualitative analysis of the target receptor in cell lysates by Western Blot as well as one representative amperometric trace recorded with the developed immunosensor for each of the tested lysates. Table 2 summarizes the results obtained and their comparison with those provided by a commercial ELISA kit, which employs the same immunoreagents and cell lysate amount to perform the determination.

**Table 2:** Determination of endogenous FGFR4 concentration (in pg μg^-1^) in different cancer cell lysates (2.5 μg). Comparison of the results provided by the developed amperometric immunosensor with those obtained using a commercial ELISA spectrophotometric kit by performing three different determinations over the same cell lysate.

Cancer cell lysate	ELISA	Magnetoimmunosensor
MCF7	(11 ± 1), RSD = 5.0%	(11.0 ± 0.3), RSD = 1.1%
MDA-MB-436	(1.8 ± 0.5), RSD = 10.0%	(1.8 ± 0.4), RSD = 9.0%
SKBR3	(13 ± 2),RSD = 7.3%	(12.9 ± 0.9), RSD = 2.7%
KM12C	(6 ± 1), RSD = 7.7%	(5.7 ± 0.3), RSD = 1.8%
KM12SM	(7 ± 2), RSD = 9.5%	(7 ± 1), RSD = 6.0%
SW480	(6.0 ± 0.9), RSD = 5.8%	(6 ± 1),RSD = 7.4%
SW620	(3.2 ± 0.5), RSD = 6.8%	(3.5 ± 0.4), RSD = 4.8%
BxPC3	(4.1 ± 0.5), RSD = 4.2%	(4.3 ± 0.2), RSD = 1.6%

Results provided with the developed immunosensor, ELISA and Western Blot demonstrated that FGFR4 was highly overexpressed in MCF7 and SKBR3 lysates in comparison with the expression levels found in MDA-MB-436 cells. Among colorectal cancer cell lines, the expression of the target receptor was larger for those lysates corresponding to highly metastatic samples (KM12SM) as compared to the low metastatic colorectal cancer cells (KM12C and SW480, SW620).

Importantly, only qualitative data for FGFR4 expression has been reported in the literature so far, highlighting the importance of the developed immunosensor to provide accurate quantitative FGFR4 data. However, the results obtained are in agreement with qualitative FGFR4 expression described in breast and colorectal cancer cells [26]. In breast cancer cells, it was recently shown that FGFR4 is highly overexpressed at mRNA level in a panel of 6 breast cancer cells from different cancer subtypes in comparison to non-transformed breast myoepithelial control cells, including MCF7 and SKBR3 [26]. At protein level, FGFR4 was more expressed in MCF7 than in SKBR3 -as it was also here observed-, with no expression in control cells. Regarding the expression of FGFR4 in colorectal cancer cells [9], an overexpression of FGFR4 at protein level in 10 out of 11 CRC cells in comparison to control cells was reported, with a larger expression of FGFR4 in highly metastatic CRC cells. Also in agreement with the results presented in this work, previous literature describes that highly metastatic KM12SM shows higher FGFR4 expression than low metastatic KM12C and SW480 CRC cells, even lesser expression having been found for BxPC3 control cells [6, 9]. Similar results for FGFR4 were observed at mRNA level [27]. No data for FGFR4 protein levels have been reported for the other cancer cells tested in our assay.

On the other hand, the excellent correlation between the FGFR4 concentrations provided with the ELISA kit and the immunosensor using MBs was found (see S5 Fig), with slope and intercept values for the correlation graph of (0.99 ± 0.02) and (0.08 ± 0.15), respectively, with the confident intervals calculated at a significance level of α = 0.05. As it can be deduced, the slope and intercept values included the unit and the zero values, respectively. It is worth to mention also here that although both methodologies provide comparative results and require the same amount of raw lysates for the determination, the significantly shorter assay time (15 vs 280 min, once the lysates have been prepared), the inherent simplicity and the compatibility with portable and cost-effective instrumentation of the electrochemical approach reported here can be claimed as important practical advantages over commercial ELISA, which uses tedious, time-consuming multistage processes and expensive detection instrumentation, making it difficult to implement it as a tool for the development of user-friendly devices to perform routine and decentralized analysis.

## Conclusions

This work describes the first biosensor developed so far for the determination of FGFR4 biomarker. The relatively simple design involving a sandwich format, magnetic immunocarriers assembled onto disposable platforms and amperometric transduction using the HRP/H_2_O_2_/HQ system, allows a highly sensitive (LOD of 48.2 pg mL^-1^) and selective determination of the target protein receptor in just 15 min using a single step protocol. The applicability of this approach has been demonstrated through the accurate determination of the endogenous content of FGFR4 in 8 different cancer cell lysates. The obtained results are in good agreement with those provided by a commercial ELISA methodology but using a simpler methodology and an 18 times lower analysis time (15 vs 280 min) once the cells lysates have been prepared. Therefore, the great analytical performance exhibited by this amperometric platform of simple and rapid operation, disposable format and the possibility to use pocket-size electrochemical transducers, can be claimed as important advantages for potential integration into portable and user-friendly point-of-care (POC) devices to perform high throughput routine determinations.

## Supporting information

S1 Fig Comparison between the amperometric responses obtained at -0.20 V with immunosensors constructed with the CAb-immobilized or non-CAb modified-MBs in the absence (white bars) or in the presence (grey bars) of 10,000 pg mL-1 FGFR4. Error bars were estimated as triple of the standard deviation (n = 3).
(PDF)

S2 Fig Dependence of the amperometric responses measured with the developed immunosensor in the absence (white bars) or in the presence of 5,000 pg mL^-1^ FGFR4 (grey bars) and the corresponding S/B ratio (•) as a function of the HOOC-MBs amount (a), CAb concentration (b), incubation time with CAb (c) incubation time with mixture (BDAb + Target FGFR4 + Strep-HRP) (d) and Strep-HRP dilution factor (e). Error bars were estimated as triple of the standard deviation (n = 3).
(PDF)

S3 Fig Control chart constructed to check the stability of b-AbC-MBs conjugates stored in filtered PBS (pH 7.5) at 4°C. S/B values corresponding to amperometric signals obtained for standard solutions containing 0.0 and 2,500 pg mL-1 FGFR4.
(PDF)

S4 Fig Calibration plot obtained with the electrochemical immunosensor for samples solutions containing 2.5 μg of raw KM12SM lysate solution supplemented with increasing amounts of the standard FGFR4 solution. Error bars are estimated as a triple that of the standard deviation (n = 3).
(PDF)

S5 Fig Comparison of the results obtained with the developed immunosensor and the ELISA methodologies. Dots in the graph correspond to (from left to right) to lysates from MDA-MB-436, SW620, BxPC3, KM12C, SW480, KM12SM, MCF7 and SKBR3 cells, respectively. Error bars are estimated as a triple that of the standard deviation (n = 3).
(PDF)
