# Supplementary figures and images for: Electrochemical sensor for rapid determination of fibroblast growth factor receptor 4 in raw cancer cell lysates
Source: PLoS One. 2017 Apr 4;12(4):e0175056. doi: 10.1371/journal.pone.0175056 (PMC5380347; doi:10.1371/journal.pone.0175056)

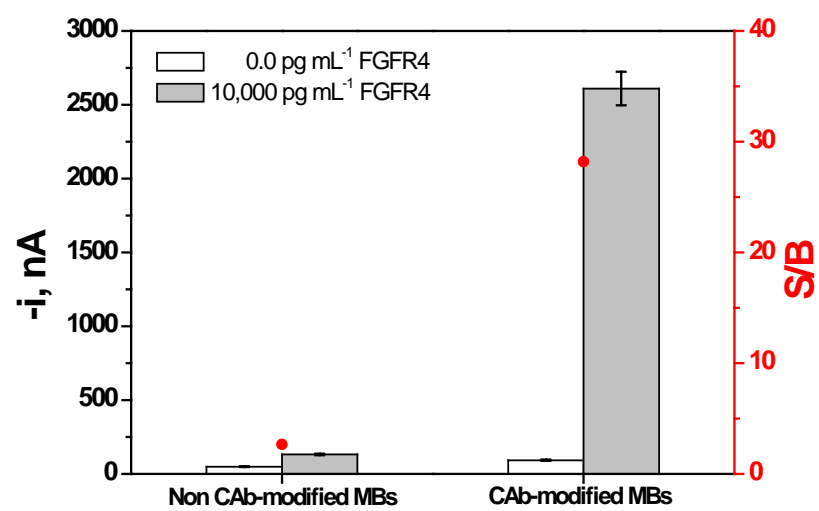

Supplement: S1 Fig — Error bars were estimated as triple of the standard deviation (n = 3). (PDF) [file pone.0175056.s001.pdf]

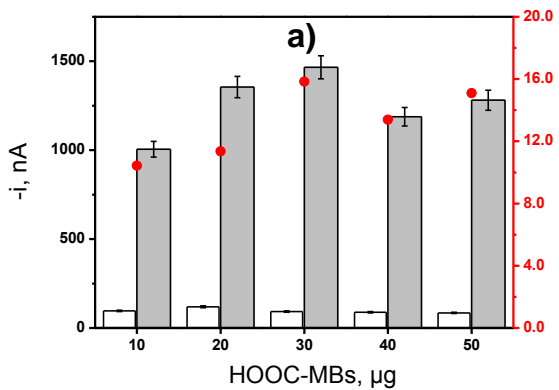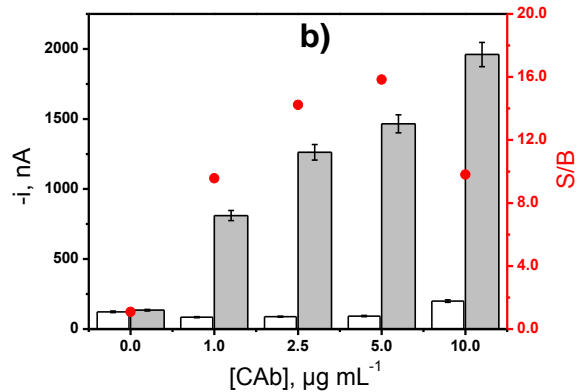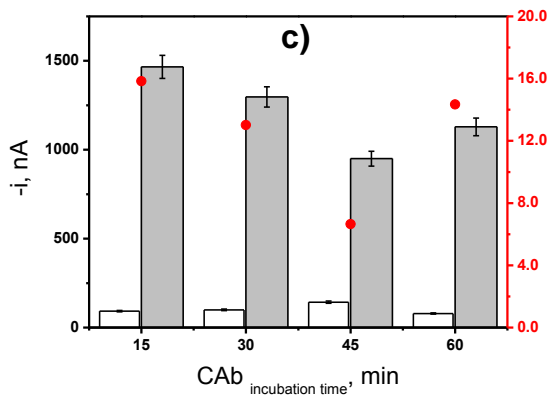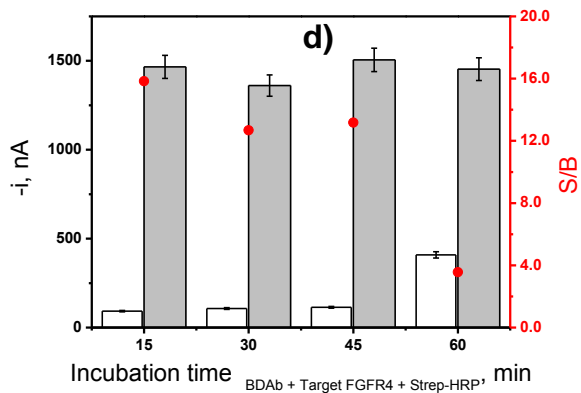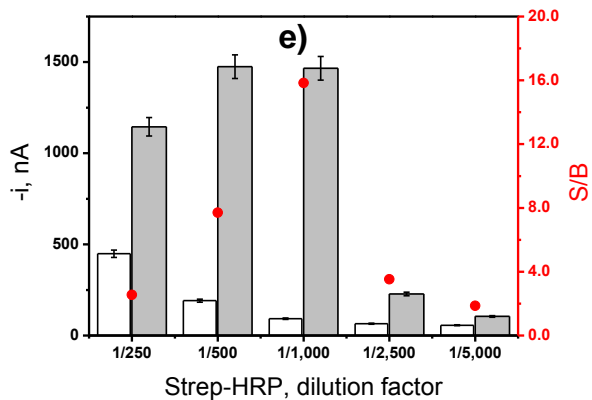

Supplement: S2 Fig — Error bars were estimated as triple of the standard deviation (n = 3). (PDF) [file pone.0175056.s002.pdf]

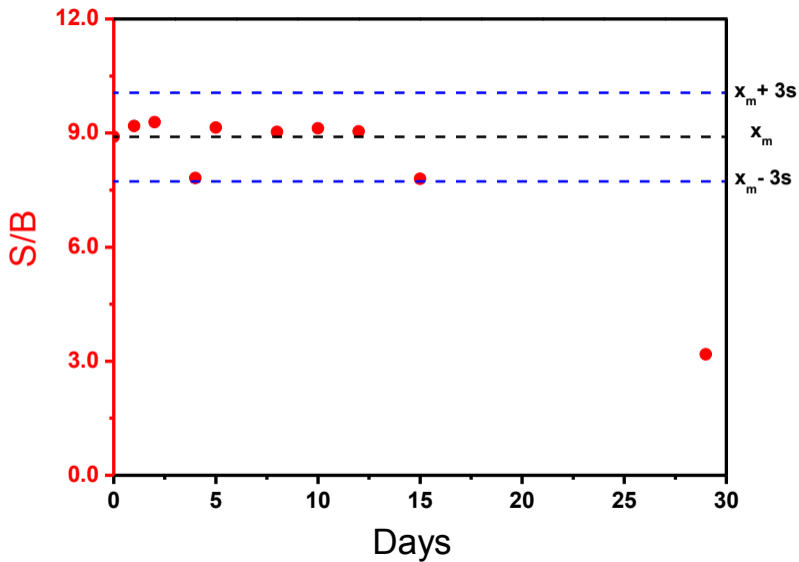

Supplement: S3 Fig — S/B values corresponding to amperometric signals obtained for standard solutions containing 0.0 and 2,500 pg mL-1 FGFR4. (PDF) [file pone.0175056.s003.pdf]

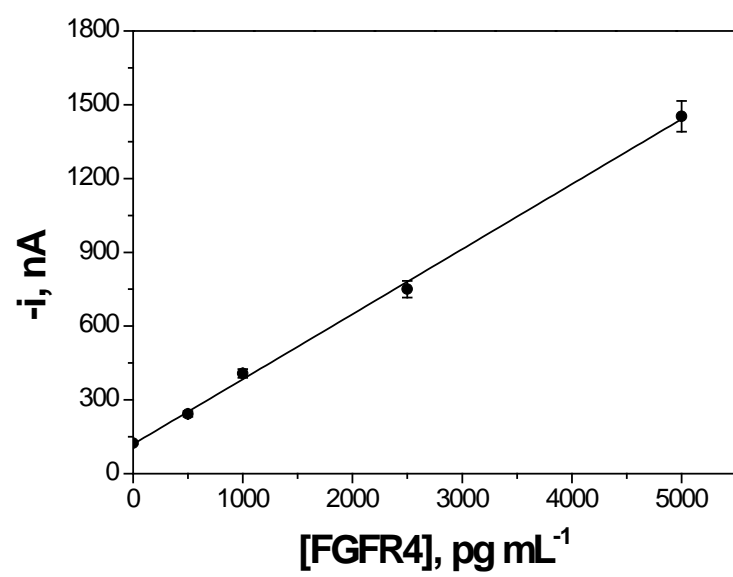

Supplement: S4 Fig — Error bars are estimated as a triple that of the standard deviation (n = 3). (PDF) [file pone.0175056.s004.pdf]

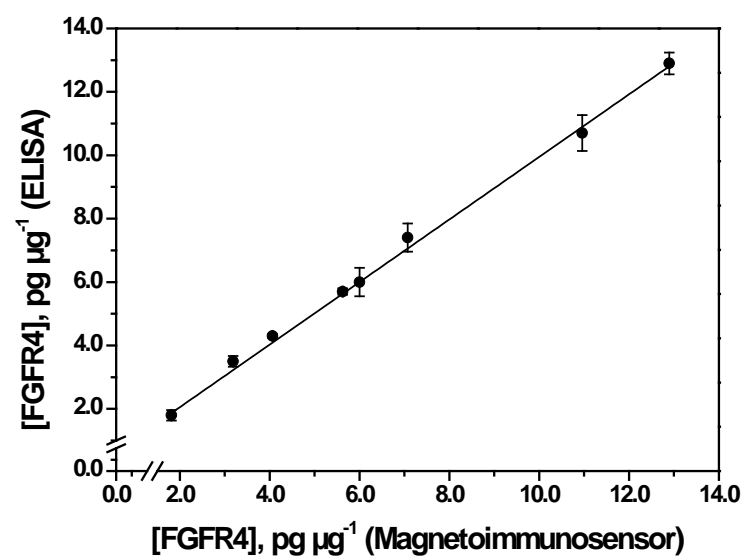

Supplement: S5 Fig — Error bars are estimated as a triple that of the standard deviation (n = 3). (PDF) [file pone.0175056.s005.pdf]
